# Developing a Prognostic Model for Primary Biliary Cholangitis Based on a Random Survival Forest Model

**DOI:** 10.7150/ijms.88481

**Published:** 2024-01-01

**Authors:** Xin-yu Fu, Ya-qi Song, Jia-ying Lin, Yi Wang, Wei-dan Wu, Jin-bang Peng, Li-ping Ye, Kai Chen, Shao-wei Li

**Affiliations:** 1 Taizhou Hospital of Zhejiang Province affiliated to Wenzhou Medical University, Linhai, Zhejiang, China.; 2 Department of Gastroenterology, Taizhou Hospital of Zhejiang Province affiliated to Wenzhou Medical University, Linhai, Zhejiang, China.; 3 Key Laboratory of Minimally Invasive Techniques & Rapid Rehabilitation of Digestive System Tumor of Zhejiang Province, Taizhou Hospital Affiliated to Wenzhou Medical University, Linhai, Zhejiang, China.; 4 Taizhou Chinese Traditional Hospital, Jiaojiang, Zhejiang, China.; 5 Institute of Digestive Disease, Taizhou Hospital of Zhejiang Province Affiliated to Wenzhou Medical University, Linhai, Zhejiang, China.

**Keywords:** Primary Biliary Cholangitis, Random Survival Forest, Prognosis, Risk assessment

## Abstract

**Background:** Primary biliary cholangitis (PBC) is a rare autoimmune liver disease with few effective treatments and a poor prognosis, and its incidence is on the rise. There is an urgent need for more targeted treatment strategies to accurately identify high-risk patients. The use of stochastic survival forest models in machine learning is an innovative approach to constructing a prognostic model for PBC that can improve the prognosis by identifying high-risk patients for targeted treatment.

**Method:** Based on the inclusion and exclusion criteria, the clinical data and follow-up data of patients diagnosed with PBC-associated cirrhosis between January 2011 and December 2021 at Taizhou Hospital of Zhejiang Province were retrospectively collected and analyzed. Data analyses and random survival forest model construction were based on the R language.

**Result:** Through a Cox univariate regression analysis of 90 included samples and 46 variables, 17 variables with p-values <0.1 were selected for initial model construction. The out-of-bag (OOB) performance error was 0.2094, and K-fold cross-validation yielded an internal validation C-index of 0.8182. Through model selection, cholinesterase, bile acid, the white blood cell count, total bilirubin, and albumin were chosen for the final predictive model, with a final OOB performance error of 0.2002 and C-index of 0.7805. Using the final model, patients were stratified into high- and low-risk groups, which showed significant differences with a P value <0.0001. The area under the curve was used to evaluate the predictive ability for patients in the first, third, and fifth years, with respective results of 0.9595, 0.8898, and 0.9088.

**Conclusion:** The present study constructed a prognostic model for PBC-associated cirrhosis patients using a random survival forest model, which accurately stratified patients into low- and high-risk groups. Treatment strategies can thus be more targeted, leading to improved outcomes for high-risk patients.

## Introduction

Primary biliary cholangitis (PBC), previously known as primary biliary cirrhosis, is a classic autoimmune liver disease characterized by destructive lymphocytic cholangitis and the presence of specific anti-mitochondrial antibodies (AMAs) 1-3. PBC leads to immune-mediated injury to biliary epithelial cells (BECs), resulting in cholestasis, progressive liver fibrosis, and a spectrum of clinical manifestations. It is a slowly progressive disease with a natural history of 10-15 years leading to end-stage liver disease 4, 5. However, high-risk cases can still progress rapidly to decompensated cirrhosis or even death 6. Ursodeoxycholic acid (UDCA) has demonstrated some efficacy in slowing the progression of the disease, but its impact is limited, especially in rapidly progressing patients 7, 8. Therefore, it is essential to promptly identify and intervene in high-risk patients to effectively manage the disease progression.

Primary biliary cholangitis (PBC) is a relatively rare autoimmune liver disease, but its global prevalence is increasing 9. Currently, there are no definitive prognostic indicators for PBC, and prognosis assessment heavily relies on regular patient follow-ups, placing a significant burden on both patients and healthcare providers 10. Accurately identifying and distinguishing high-risk from low-risk patients and intervening in high-risk cases could effectively improve outcomes. However, there is currently a lack of reported PBC prognostic models, leaving a void in this critical clinical area. Developing an effective prognostic model holds clinical value and could be beneficial in the management of PBC.

Machine learning algorithms, such as random forest plots, have shown promising potential in medicine for various applications, including disease diagnoses, patient outcome prediction, and drug discovery 11-13. In the context of PBC, a randomized survival forest model in machine learning can be used to construct a prognostic model for PBC-associated cirrhosis. This model can accurately identify high-risk patients and enable targeted treatment. The randomized survival forest model is a powerful tool for constructing prognostic models in survival analyses due to its ability to handle complex and high-dimensional datasets and its resistance to overfitting. The proposed use of machine learning in the prognostic analysis of PBC represents an innovative approach that can potentially improve patient outcomes and lead to better management of this disease.

## Methods

### Study design and patients

This study retrospectively collected and analyzed the clinical data and follow-up information of 90 patients with PBC-related cirrhosis diagnosed in Taizhou Hospital, Zhejiang Province, China, from January 2011 to December 2021. This study was approved by the Ethics Committee of Taizhou Hospital of Wenzhou Medical University (approval number: K20220234).

The inclusion criteria were as follows: 1) a clear diagnosis of PBC; 2) progression to cirrhosis on the basis of PBC, 3) and complete follow-up data.

The exclusion criteria were as follows: 1) short follow-up (<3 months) or lost to follow-up during the follow-up process; 2) alcoholic liver disease, nonalcoholic steatohepatitis, viral hepatitis, autoimmune hepatitis, or other liver diseases as well as tumors or severe cardiac, renal, respiratory, hematologic, or mental health disorders; 3) and patients who received a liver transplant before enrollment or during the follow-up period.

The diagnostic criteria for PBC were as follows: 1) serum AMA titer ≥ 1:40; 2) unexplained ALP elevation ≥ 1.5 times the upper normal value for more than 24 weeks; 3) and liver tissue compatibility, especially nonsuppurative cholangitis and interlobular bile duct injury 14. The diagnosis of cirrhosis was based on pathological, ultrasound, or radiological signs in the liver. Decompensated cirrhosis was defined by the development of cirrhotic ascites, ruptured esophageal and gastric varices bleeding, hepatic encephalopathy, and other complications.

### Inclusion of variables

This study included the following variables: age; gender; stage of cirrhosis; complications of cirrhosis (ascites, esophageal and gastric varices, ruptured and bleeding esophageal and gastric varices, infections, hepatic encephalopathy); concomitant diseases (autoimmune diseases, gallbladder diseases, osteoporosis); serological tests at the diagnosis, including white blood cells (WBCs), neutrophils (Ns), lymphocytes (Ls), platelets (Plts), and levels of C-reactive protein (CRP), alanine aminotransferase (ALT), aspartate aminotransferase (AST), alkaline phosphatase (ALP), gamma-glutamyl transferase (GGT), bilirubin (BIL), albumin (Alb), cholinesterase (ChE), serum creatinine (CR), PT-INR, serum Na, triglycerides, cholesterol, low-density lipoprotein cholesterol, complement C3, complement C4, IgM, alpha-fetoprotein, carcinoembryonic antigen, CA199, CA125, CA153, hyaluronic acid (HA), laminin (LN), type IV collagen, type III precollagen N-terminal peptide (PIIINP), antinuclear antibody series (ANA, AMA, AMAM2, SSA, SSA52, SSB anti-sp100, anti-gp210, anti-dsDNA antibody, anti-adhesion point protein B antibody, anti-RNP antibody); and whether the patient was readmitted within 1 month, 3 months, or 6 months.

### Data analyses and model construction

Variable screening was conducted using COX regression, where variables with p-values <0.1 were selected for inclusion in the random survival forest model. The model's variables were further screened, and the top five variables with the highest scores were chosen as the final variables for model construction. An evaluation of the models was performed using the C-index, out-of-bag (OOB) error, and receiver operating characteristic (ROC) curve area. All data analyses and model constructions for the random survival forest were performed using the R language (version 4.2.3).

The C-index is a widely used measure of a model's predictive accuracy for survival data 15. It ranges from 0 to 1, where 1 indicates perfect predictive accuracy, and 0.5 indicates random prediction. The OOB error is a method used to estimate a model's prediction error without requiring a separate validation set 16. It estimates the error by comparing the predictions of each bootstrap sample to the actual outcomes of the observations that were not included in the sample. Both the C-index and OOB error were used to evaluate the performance of the random survival forest models in the present study.

## Results

**Figure 1:**
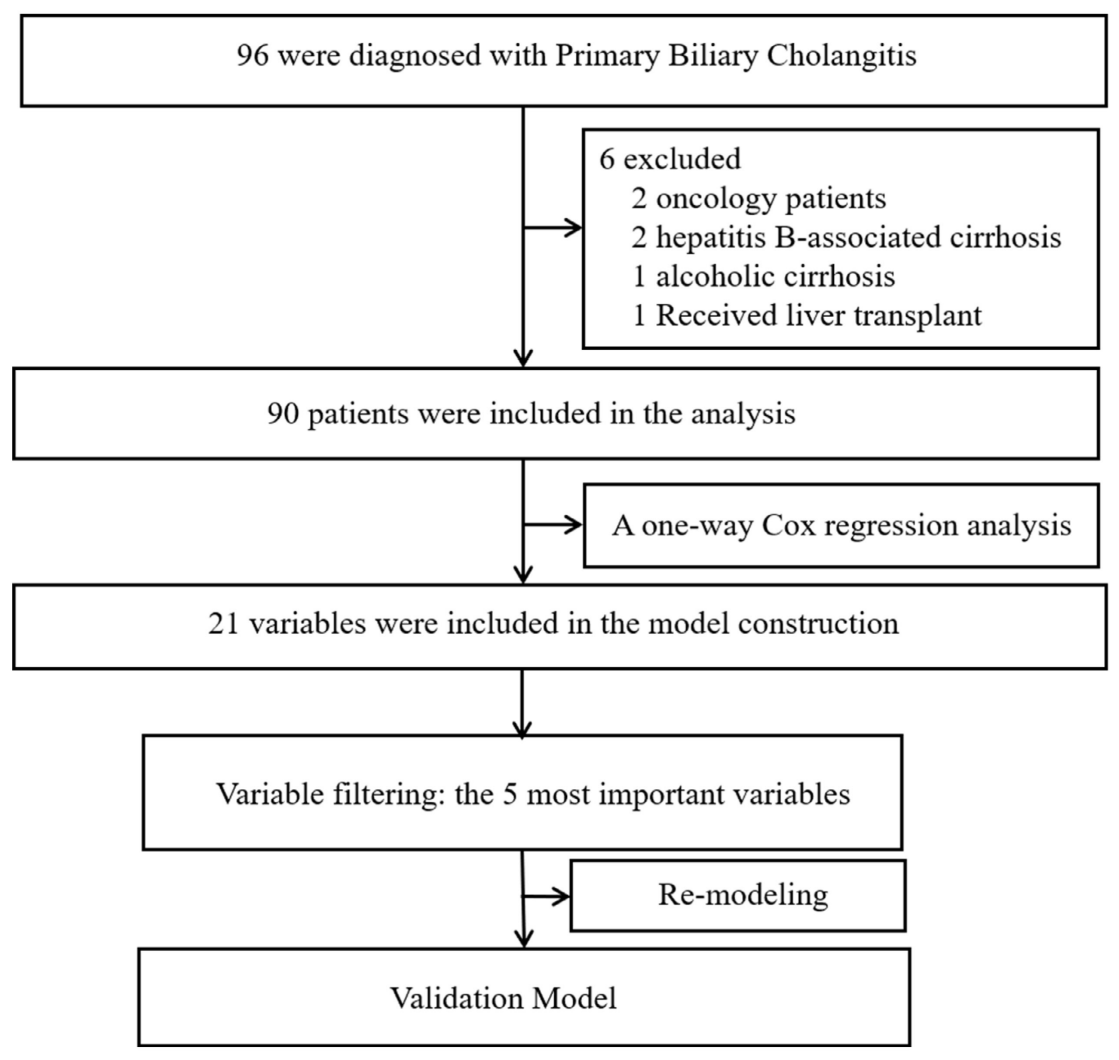
Study flow chart.

Ninety patients diagnosed with PBC-related liver cirrhosis were included in the analysis after excluding six patients (two with tumors, two with hepatitis B-related liver cirrhosis, one with alcoholic liver cirrhosis, and one who underwent liver transplantation during follow-up). Among the included patients, the mean age was 59.19±11.45 years old, with 77 females and 13 males. There are 32 cases (35.6%) of compensatory cirrhosis and 58 cases (54.4%) of decompensated cirrhosis. Among the patients, 52 have concurrent ascites, 19 have concurrent esophageal and gastric varices, 10 have concurrent infection, and 2 have concurrent hepatic encephalopathy. At the end of the follow-up period, 19 patients had passed away, while 71 remained alive. The median survival time was 35 months, and the mean survival time was 42.53 ± 32.61 months. Figure 1 shows the flow chart of the data analyses.

A one-way Cox regression analysis was conducted to evaluate the variables included in the study. In order to ensure the inclusion of an adequate number of variables, selections were made for model construction based on p-values <0.1. The following variables were found to be significant according to a univariate regression analysis: "jaundice", "spider nevus", "SSA", "leukocyte", "lymphocytes", "CRP", "AST", "bilirubin", "bile acid", "albumin", "cholinesterase", "Na", "LDL", "PT", "INR", "Complement C3", "Complement C4", "hyaluronic acid", "laminin", "type IV collagen", and "type III precollagen N-terminal peptide". Table 1 displays the corresponding p values.

**Figure 2:**
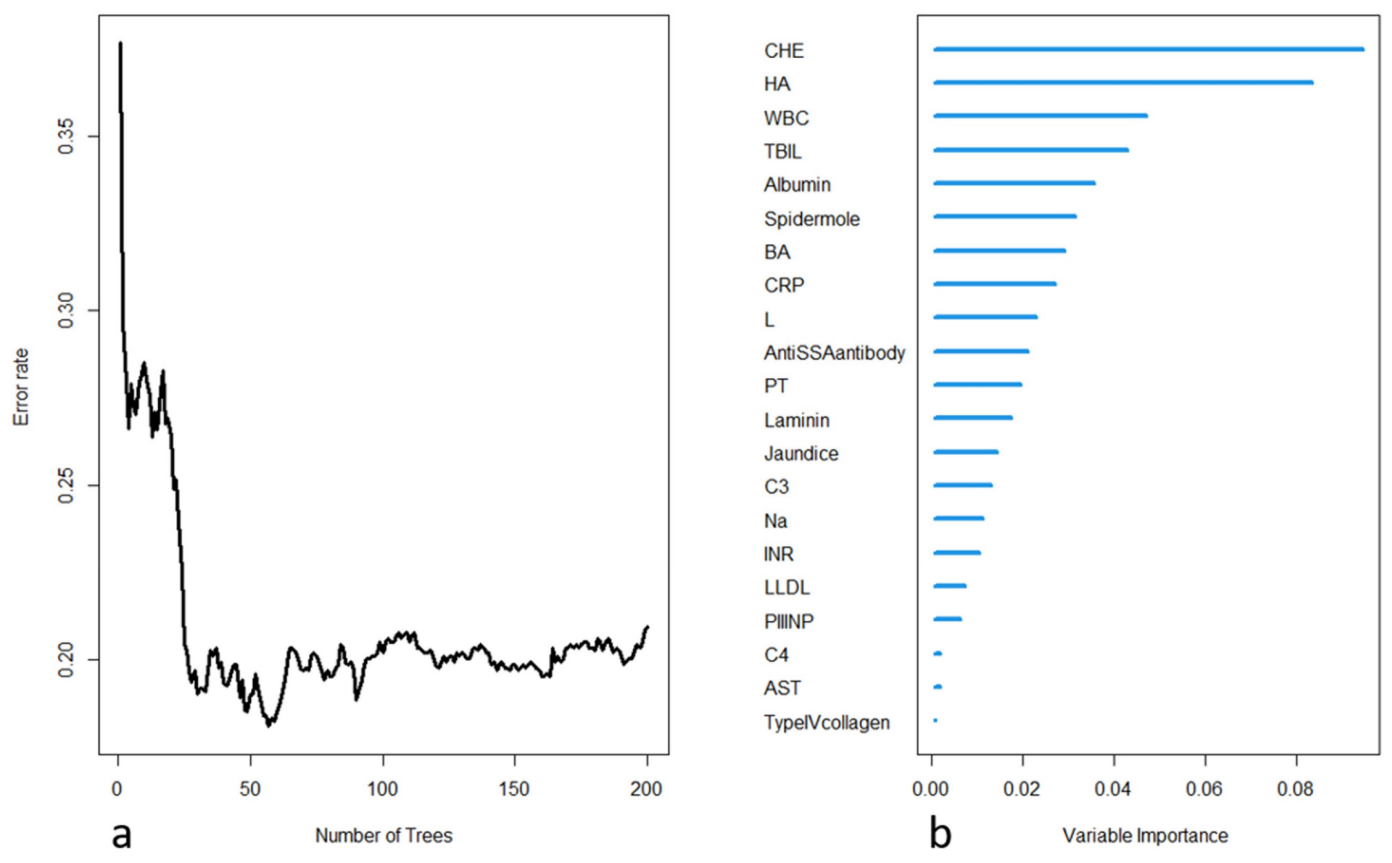
(a) Process of model training in the random forest, with the error rate decreasing as the number of trees increases; (b) importance ranking of each variable in the model output.

The selected variables were included in the construction of a random survival forest model. Two hundred decision trees were built, with 15 terminal nodes and 5 selected variables in each tree. The OOB requested performance error was 0.2094, indicating a model error rate of 20.94%. Internal validation was performed using K-fold cross-validation, and the resulting C-index was 0.8182, indicating a model accuracy of 81.82%. The model construction process is depicted in Figure 2. Further variable selection was performed using the model, and the top five variables in terms of importance were selected as the final variables to be included in the random survival forest model. Table 2 shows the differences in the death and survival groups for the five indicators that were finally included in the analysis.

**Figure 3:**
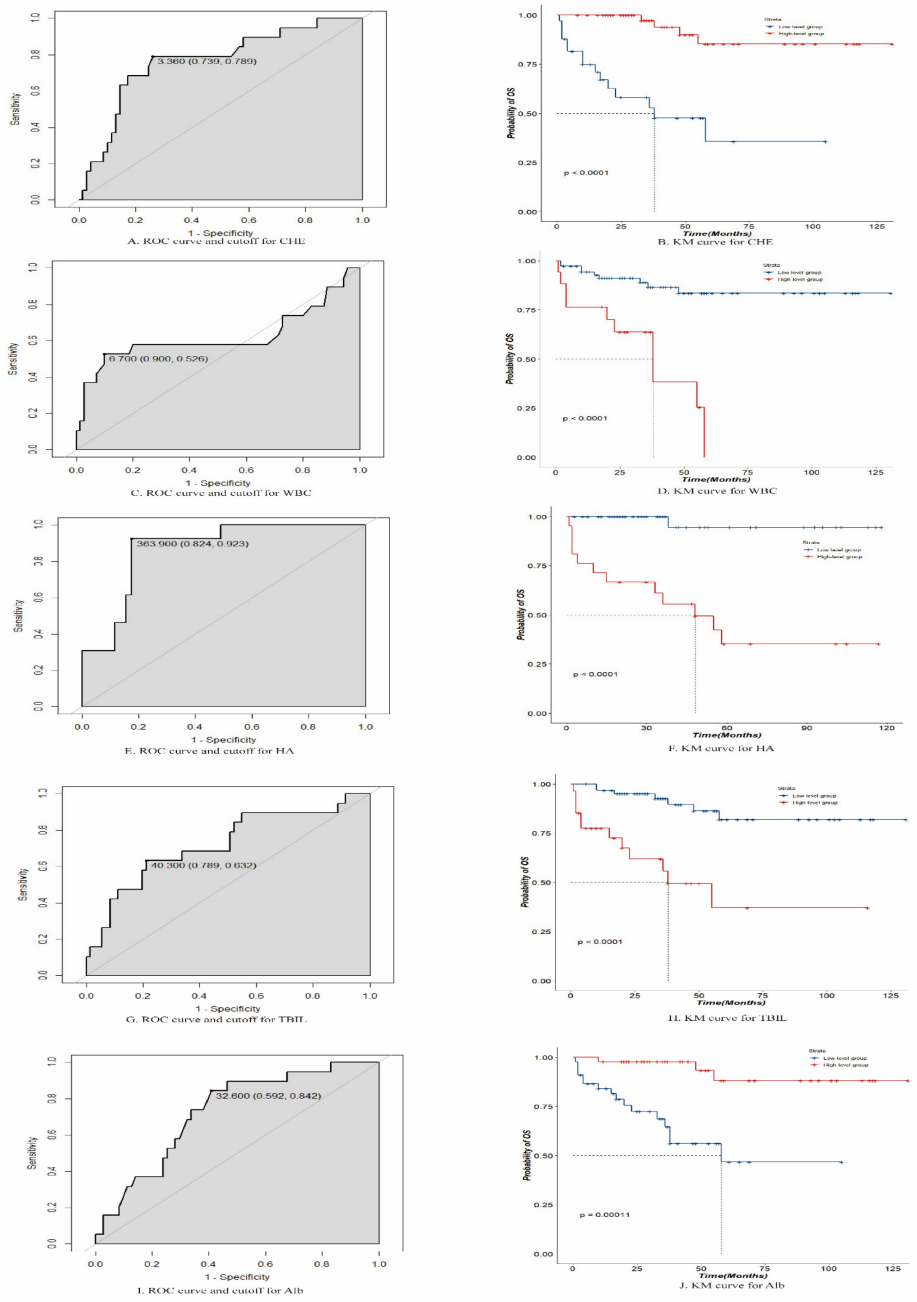
Receiver operating characteristic and Kaplan-Meier curves for cholinesterase (CHE), white blood cells (WBCs), hyaluronic acid (HA), total bilirubin (TBIL), and albumin (Alb).

The final variables included in the random survival forest model were " CHE", "HA", "WBC count", "total BIL (TBIL)", and "Alb". Using an ROC analysis, the optimal threshold values were calculated for these five variables, and patients were divided into high- and low-level groups based on these thresholds. The optimal threshold for CHE was 3.360 (confidence interval [CI]: 0.739-0.789), with patients above this threshold classified as high-level and those below it classified as low-level. The optimal threshold for HA was 363.900 (CI: 0.824-0.923), with patients above this threshold classified as high-level and those below it classified as low-level. The optimal threshold for the WBC count was 6.700 (CI: 0.526-0.900), with patients above this threshold classified as high level and those below this threshold classified as low level. The optimal threshold for TBIL was 40.300 (CI: 0.632-0.789), with patients above this threshold classified as high-level and those below it classified as low-level. The optimal threshold for Alb was 32.600, with patients above this threshold classified as high-level and those below it classified as low-level. The survival impact of these groups was demonstrated through a Kaplan-Meier analysis, as depicted in Figure 3.

**Figure 4:**
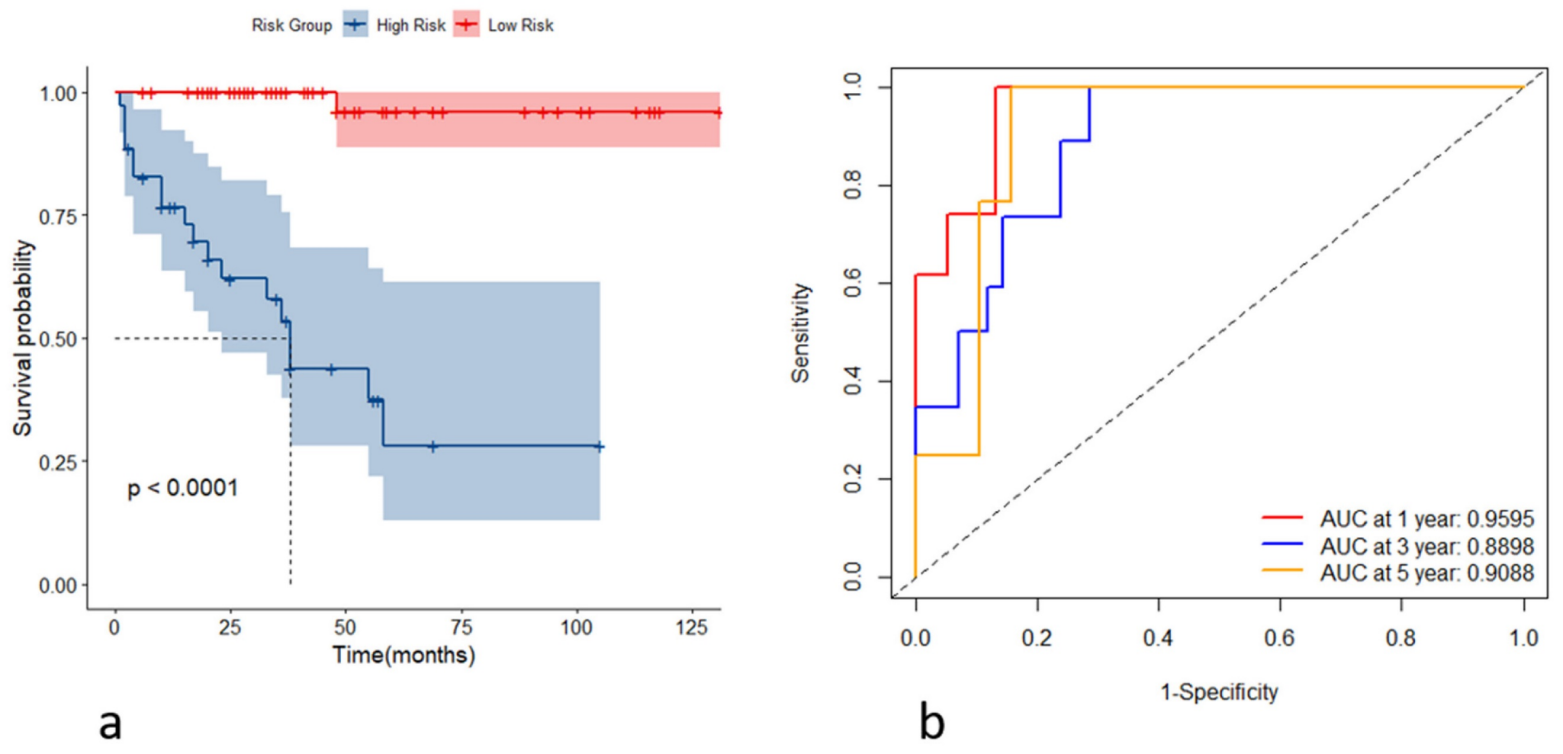
(a) Kaplan-Meier curves for the high- and low-risk groups stratified by the risk scores; (b) receiver operating characteristic curves of the model's predictive performance at the first, third, and fifth years.

**Figure 5:**
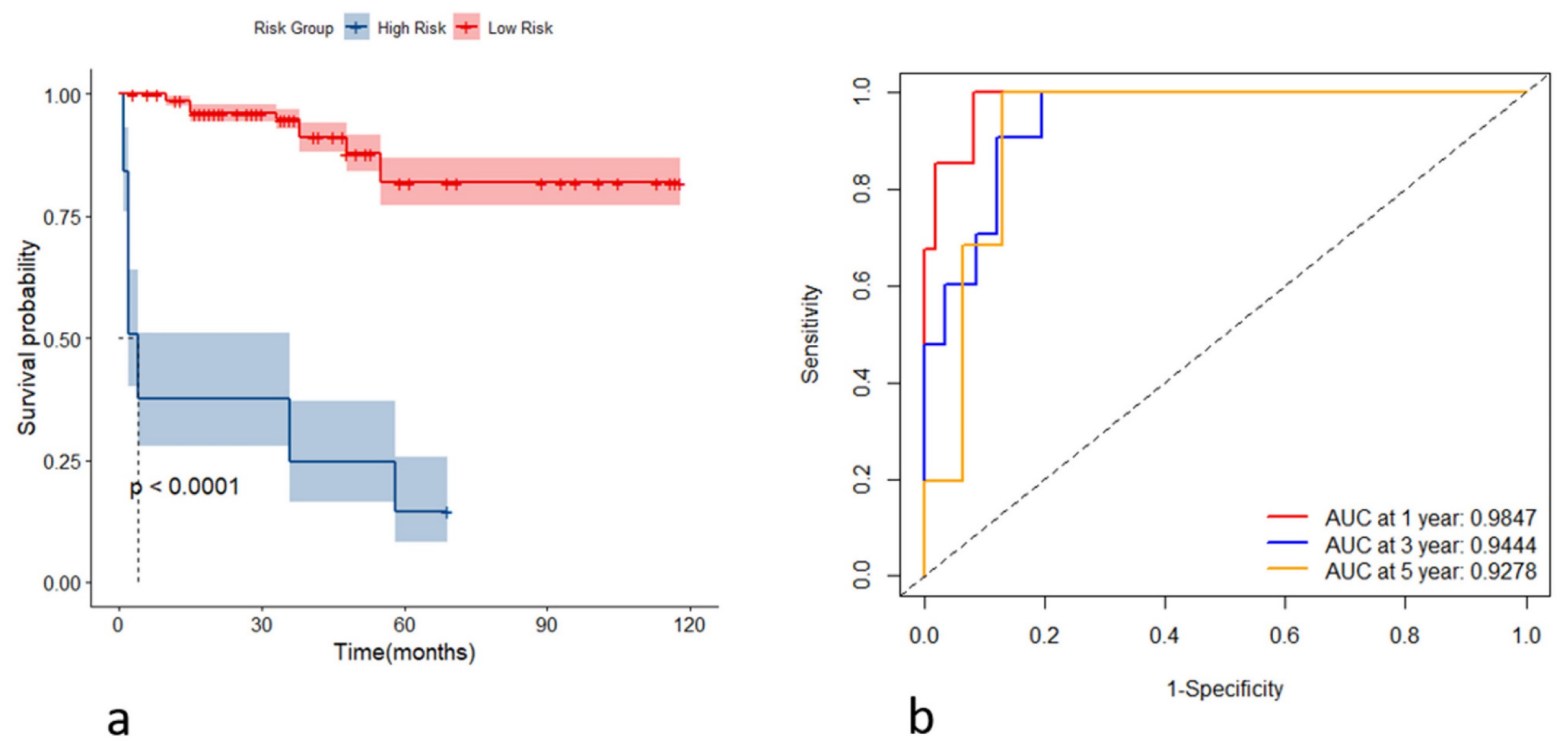
(a) Kaplan-Meier curves of the high- and low-risk group based on the risk score in the testing set; (b) receiver operating characteristic curves of the model for predicting the outcome at the first, third, and fifth years in the testing set.

Incorporating the variables CHE, WBC, TBIL, HA, and Alb, we constructed stochastic survival forest models. We utilized 200 decision trees with a minimum branch of 15, and the OOB requested performance error was 0.2002, indicating an error rate of 20.02%, with a C-index of 0.7805, indicating an accuracy rate of 78.05%, using internal k-fold cross-validation. The risk score was calculated by the model, and we segregated patients into high- and low-risk groups based on their risk score. We then plotted the survival curves to evaluate the model's predictive effect. Furthermore, we assessed the predictive efficacy of the model in predicting survival at years 1, 3 and 5 using ROC, and the results are expressed as the area under the curve (AUC). The AUCs for year 1, year 3, and year 5 were 0.9595, 0.8898, and 0.9088, respectively, as shown in Figure 4. To further validate the model's effect, we used a put backable random resampling method to construct a test set by sampling 900 times. We then validated the constructed model on the test set, calculated the sub risk scores, and classified patients into high- and low-risk groups. Finally, we calculated the predictive effects using an ROC analysis for year 1, year 3, and year 5. The AUCs for years 1, 3, and 5 were 0.9847, 0.9444, and 0.9278, respectively, as shown in Figure 5.

## Discussion

PBC is a rare autoimmune liver disease characterized by inflammation and progressive destruction of interlobular bile ducts and biliary stasis causing debilitating fatigue and pruritus, ultimately leading to cirrhosis and death 17, 18. We constructed a prognostic model for PBC-related cirrhosis using a stochastic survival forest model in machine learning. Compared to the traditional multifactor regression model, the machine learning model exhibits higher stability. To avoid overfitting caused by a large number of variables during model training, we selected CHE, WBC, TBIL, HA, and Alb as the final training variables through Cox regression and model training. The final model achieved an out-of-bag (OOB) value of 0.2002 and a C-index of 0.7805, comparable to the initially constructed model, demonstrating good accuracy and error rates. Internal validation showed an OOB value of 0.2195 and a C-index of 0.7805, indicating good stability. The area under the curve (AUC) for the first, third, and fifth-year predictions were 0.9595, 0.8898, and 0.9088, respectively. The prediction model showed superior prediction results.

PBC is a classic autoimmune liver disease characterized by ongoing and specific damage to the bile ducts, resulting in impaired bile flow and progressive liver injury, ultimately leading to the development of cirrhosis 2. Currently, there are no specific markers that can reflect the progression and prognosis of PBC, highlighting the urgent need for an effective predictive model 2. In this study, we employed a random survival forest model and identified five highly correlated variables: cholinesterase, hyaluronic acid, albumin, total bilirubin, and white blood cells. Cholinesterase, as one of the synthetic products in the liver, catalyzes the hydrolysis of cholinester compounds in the human body, breaking them down into acetic acid and choline 19. Numerous studies have reported increased secretion of cholinesterase in the serum of PBC patients, likely due to enhanced synthesis and secretion in response to liver inflammation and injury. Whether cholinesterase is involved in the generation of anti-mitochondrial antibodies (AMAs) in PBC remains to be further elucidated. Although the precise mechanisms are still unclear, the sustained elevation of cholinesterase in PBC patients suggests its crucial role in the immunopathogenesis of PBC occurrence and development. Serum transparency inositol phosphate can reflect the endothelial function and level of damage in the liver, and it is clinically used to evaluate the degree of liver fibrosis. In patients with PBC, cirrhosis is an inevitable stage, and transparency inositol phosphate can reflect the extent of cirrhosis, which has some significance for the prognosis of PBC 2, 20-22. Albumin and bilirubin, as the most representative products of liver synthetic and metabolic functions, can indicate the functional level of the liver and provide certain hints regarding the progression of PBC 23, 24. White blood cells (WBC), as the primary immune cells in the human body, participate in the majority of immune reactions and their levels can reflect the body's inflammatory state to a certain extent. Considering that PBC is characterized by chronic liver inflammation, WBC count can serve as an indicator of PBC severity 25. In conclusion, our findings highlight the significance of these five variables in the pathogenesis and progression of PBC, providing valuable insights for the development of an effective predictive model.

The random survival forest model has been widely applied and shown to exhibit better stability and accuracy compared to traditional regression models. It has been used in various prognostic models, including predicting gene phenotypes and prognoses for different diseases 26-28. A study utilizing SEER data to construct multiple prognostic models for pancreatic cancer demonstrated that the random survival forest outperformed Cox regression (C-index of 0.670) and neural networks (C-index of 0.700) with a higher C-index of 0.723^29^. Random survival forests seem to yield better predictive performance for survival data. Moreover, random survival forests have demonstrated high stability in biomarker validation. There have been reports of their use in identifying novel biomarkers for predicting event-free survival in high-risk pediatric acute lymphoblastic leukemia, enabling the differentiation of high-risk patients and improving prognosis 30. In our research, we employed the random survival forest algorithm to construct a prognostic model for PBC and achieved high predictive accuracy. Additionally, this model accurately identifies individuals at high risk, which holds clinical significance. Close follow-up and intervention are necessary for PBC patients classified as high risk to monitor disease progression. Furthermore, liver transplantation appears to be a more effective treatment for high-risk patients, providing valuable insights into prioritizing liver transplantation.

There are some limitations to our study. The clinical data used in our study were all from Taizhou Hospital in Zhejiang Province, China, which may have some biases. In addition, the small sample size may have led to overfitting during the model training process, which may have affected the accuracy of the model's predictions. Our model incorporates five indicators as predictive models, which cannot be compared to traditional single indicators like ALP. This limitation necessitates further data validation in future research. Furthermore, due to the limited sample size, only internal validation was performed in this study, and external validation was not conducted, which limits the comprehensive evaluation of the model's accuracy. However, PBC itself is a rare autoimmune liver disease, with an incidence rate of approximately 20-40 cases per 100,000 people, and the sample size is much smaller than that of common diseases 31. Additionally, as the largest medical center in Taizhou City, Taizhou Hospital possesses strong medical resources and frequently receives referrals of patients with rare diseases from neighboring areas. Therefore, the bias in our dataset is within an acceptable range of error. The model trained based on a small sample size is also more suitable for small-sample data.

PBC is a rare disease with a small sample size, making it suitable for our model. Our random survival forest model was able to accurately distinguish high- and low-risk PBC patients and predict their prognosis, generating clinical value.

## Figures and Tables

**Table 1:** Results of Cox single-factor analyses

Variable	Beta	HR	Lower_95	Upper_95	P.value	Global.pval
Anti SSA antibody	1.270	3.580	1.430	8.920	0.006	0.010
Jaundice	1.540	4.690	1.780	12.400	0.002	0.001
Spider mole	1.400	4.040	1.340	12.200	0.013	0.032
Hyaluronic Acid	0.003	1.003	1.002	1.004	<0.001	<0.001
INR	1.730	5.640	2.660	11.900	<0.001	<0.001
PT	0.204	1.230	1.120	1.340	<0.001	<0.001
Bilirubin	0.008	1.010	1.000	1.010	<0.001	0.001
Bile acids	0.008	1.010	1.000	1.010	<0.001	0.001
White blood cells	0.203	1.220	1.100	1.370	<0.001	0.001
Cholinesterase	-0.574	0.563	0.411	0.771	<0.001	0.000
CRP	0.040	1.040	1.020	1.070	0.001	0.006
Albumin	-0.123	0.885	0.821	0.954	0.001	0.001
Na	-0.203	0.816	0.696	0.957	0.012	0.013
AST	0.001	1.001	1.000	1.002	0.013	0.046
Type IV collagen	0.006	1.010	1.000	1.010	0.034	0.076
C3	-1.510	0.220	0.050	0.979	0.047	0.043
PⅢNP	0.005	1.010	1.000	1.010	0.062	0.102
Laminin	0.007	1.010	1.000	1.010	0.065	0.106
Lymphocytes	0.549	1.730	0.963	3.110	0.067	0.080
L-LDL	0.209	1.230	0.984	1.540	0.069	0.104
C4	-5.790	0.003	0.000	2.720	0.095	0.076

PIIINP: Type III precollagen N terminal peptide; Beta: an important parameter that measures the effect of each explanatory variable on the hazard (or risk) rate; HR: hazard ratio, a statistical measure used in survival analyses to compare the probability of an event occurring between two groups, with HR > 1 indicating an increased risk and HR < 1 indicating a decreased risk; Global.pval: a statistic that evaluates the statistical significance of the overall association between a set of predictor variables and a response variable in a regression model.

**Table 2:** Differences in selected variables between the death and survival groups

Variable	Alive, N = 71^1^	Death, N = 19^1^	p value^2^
**Age**	59 (52, 68)	58 (54, 70)	0.800
**Gender**			0.500
Female	62 (87%)	15 (79%)	
Male	9 (13%)	4 (21%)	
**WBC**	4.25 (3.00, 5.20)	6.90 (2.80, 10.00)	0.110
**TBIL**	22.00 (15.32, 46.35)	93.90 (52.38, 275.85)	0.003
**Alb**	34.85 (31.60, 38.92)	29.45 (25.77, 32.10)	0.003
**HA**	182.80(121.00, 258.00)	526.30 (429.90, 1104.00)	0.003
**CHE**	4.56 (3.32, 6.11)	2.43 (2.03, 3.31)	<0.001
**Time**	37.00 (22.00, 63.00)	17.00(4.00, 37.00)	<0.001

^1^Median (interquartile range); n (%);^2^Wilcoxon rank sum test; Fisher's exact test

## References

[1] Nevens F, Trauner M, Manns MP (2023). Primary biliary cholangitis as a roadmap for the development of novel treatments for cholestatic liver diseases(†). J Hepatol.

[2] Gulamhusein AF, Hirschfield GM (2020). Primary biliary cholangitis: pathogenesis and therapeutic opportunities. Nat Rev Gastroenterol Hepatol.

[3] Tanaka A (2021). Current understanding of primary biliary cholangitis. Clin Mol Hepatol.

[4] Harms MH, Lammers WJ, Thorburn D, Corpechot C, Invernizzi P, Janssen HLA (2018). Major Hepatic Complications in Ursodeoxycholic Acid-Treated Patients With Primary Biliary Cholangitis: Risk Factors and Time Trends in Incidence and Outcome. Am J Gastroenterol.

[5] Mayo MJ (2022). Mechanisms and molecules: What are the treatment targets for primary biliary cholangitis?. Hepatology.

[6] Cordell HJ, Fryett JJ, Ueno K, Darlay R, Aiba Y, Hitomi Y (2021). An international genome-wide meta-analysis of primary biliary cholangitis: Novel risk loci and candidate drugs. J Hepatol.

[7] Shang Y, Leung PSC, Gershwin ME, Han Y (2022). Primary biliary cholangitis: personalized medicine for optimal therapeutic opportunities. Sci Bull (Beijing).

[8] Lleo A, Leung PSC, Hirschfield GM, Gershwin EM (2020). The Pathogenesis of Primary Biliary Cholangitis: A Comprehensive Review. Semin Liver Dis.

[9] Trivedi PJ, Hirschfield GM (2021). Recent advances in clinical practice: epidemiology of autoimmune liver diseases. Gut.

[10] Shah RA, Kowdley KV (2020). Current and potential treatments for primary biliary cholangitis. Lancet Gastroenterol Hepatol.

[11] Haug CJ, Drazen JM (2023). Artificial Intelligence and Machine Learning in Clinical Medicine, 2023. N Engl J Med.

[12] Carracedo-Reboredo P, Liñares-Blanco J, Rodríguez-Fernández N, Cedrón F, Novoa FJ, Carballal A (2021). A review on machine learning approaches and trends in drug discovery. Comput Struct Biotechnol J.

[13] Hu J, Szymczak S (2023). A review on longitudinal data analysis with random forest. Brief Bioinform.

[14] Younossi ZM, Bernstein D, Shiffman ML, Kwo P, Kim WR, Kowdley KV (2019). Diagnosis and Management of Primary Biliary Cholangitis. Am J Gastroenterol.

[15] Zhao B, Gabriel RA, Vaida F, Lopez NE, Eisenstein S, Clary BM (2020). Predicting Overall Survival in Patients with Metastatic Rectal Cancer: a Machine Learning Approach. J Gastrointest Surg.

[16] Bylander T (2002). Estimating generalization error on two-class datasets using out-of-bag estimates. Machine Learning.

[17] Nevens F, Andreone P, Mazzella G, Strasser SI, Bowlus C, Invernizzi P (2016). A Placebo-Controlled Trial of Obeticholic Acid in Primary Biliary Cholangitis. N Engl J Med.

[18] Lammers WJ, van Buuren HR, Hirschfield GM, Janssen HL, Invernizzi P, Mason AL (2014). Levels of alkaline phosphatase and bilirubin are surrogate end points of outcomes of patients with primary biliary cirrhosis: an international follow-up study. Gastroenterology.

[19] Korabecny J, Soukup O (2021). Cholinesterase Research. Biomolecules.

[20] Melter M, Rodeck B, Kardorff R, Hoyer PF, Petersen C, Ballauff A (2000). Progressive familial intrahepatic cholestasis: partial biliary diversion normalizes serum lipids and improves growth in noncirrhotic patients. Am J Gastroenterol.

[21] Zhang LF, Wang XH, Zhang CL, Lee J, Duan BW, Xing L (2022). Sequential Nano-Penetrators of Capillarized Liver Sinusoids and Extracellular Matrix Barriers for Liver Fibrosis Therapy. ACS Nano.

[22] Loomba R, Adams LA (2020). Advances in non-invasive assessment of hepatic fibrosis. Gut.

[23] China L, Freemantle N, Forrest E, Kallis Y, Ryder SD, Wright G (2021). A Randomized Trial of Albumin Infusions in Hospitalized Patients with Cirrhosis. N Engl J Med.

[24] Perez Ruiz de Garibay A, Kortgen A, Leonhardt J, Zipprich A, Bauer M (2022). Critical care hepatology: definitions, incidence, prognosis and role of liver failure in critically ill patients. Crit Care.

[25] Mafra K, Nakagaki BN, Castro Oliveira HM, Rezende RM, Antunes MM, Menezes GB (2019). The liver as a nursery for leukocytes. J Leukoc Biol.

[26] Sapir-Pichhadze R, Kaplan B (2020). Seeing the Forest for the Trees: Random Forest Models for Predicting Survival in Kidney Transplant Recipients. Transplantation.

[27] Ning S, Li H, Qiao K, Wang Q, Shen M, Kang Y (2020). Identification of long-term survival-associated gene in breast cancer. Aging (Albany NY).

[28] van der Heide EMM, Veerkamp RF, van Pelt ML, Kamphuis C, Athanasiadis I, Ducro BJ (2019). Comparing regression, naive Bayes, and random forest methods in the prediction of individual survival to second lactation in Holstein cattle. J Dairy Sci.

[29] Wang Z, Chen W, Zuo L, Xu M, Wu Y, Huang J (2022). The Fibrillin-1/VEGFR2/STAT2 signaling axis promotes chemoresistance via modulating glycolysis and angiogenesis in ovarian cancer organoids and cells. Cancer Commun (Lond).

[30] Bohannan ZS, Coffman F, Mitrofanova A (2022). Random survival forest model identifies novel biomarkers of event-free survival in high-risk pediatric acute lymphoblastic leukemia. Comput Struct Biotechnol J.

[31] Cazzagon N, Gonzalez-Sanchez E, El-Mourabit H, Wendum D, Rainteau D, Humbert L (2023). Protective potential of the gallbladder in primary sclerosing cholangitis. JHEP Rep.

